# Disposable surgical face masks for preventing surgical wound infection in clean surgery

**DOI:** 10.1590/S1516-31802012000400014

**Published:** 2012-09-04

**Authors:** Allyson Lipp, Peggy Edwards

## Abstract

**BACKGROUND:**

Surgical face masks were originally developed to contain and filter droplets containing microorganisms expelled from the mouth and nasopharynx of healthcare workers during surgery, thereby providing protection for the patient. However, there are several ways in which surgical face masks could potentially contribute to contamination of the surgical wound, e.g. by incorrect wear or by leaking air from the side of the mask due to poor string tension.

**OBJECTIVES:**

To determine whether disposable surgical face masks worn by the surgical team during clean surgery prevent postoperative surgical wound infection.

**SEARCH METHODS:**

We searched The Cochrane Wounds Group Specialised Register (searched 14 September 2011); The Cochrane Central Register of Controlled Trials (CENTRAL) (The Cochrane Library 2011, Issue 3); Ovid MEDLINE (2008 to August Week 5 2011); Ovid MEDLINE (In-Process &Other Non-Indexed Citations September 13, 2011); Ovid EMBASE (2008 to 2011 Week 35); and EBSCO CINAHL (2008 to 9 September 2011).

**SELECTION CRITERIA:**

Randomized controlled trials (RCTs) and quasi-randomized controlled trials comparing the use of disposable surgical masks with the use of no mask.

**DATA COLLECTION AND ANALYSIS:**

Two review authors extracted data independently.

**MAIN RESULTS:**

Three trials were included, involving a total of 2113 participants. There was no statistically significant difference in infection rates between the masked and unmasked group in any of the trials.

**AUTHORS’ CONCLUSIONS:**

From the limited results it is unclear whether the wearing of surgical face masks by members of the surgical team has any impact on surgical wound infection rates for patients undergoing clean surgery.

**This is the abstract of a Cochrane review published in the Cochrane database of systematic reviews (CDSR) 2012, issue 1, art. No.: CD002929. DOI:** 10.1002/14651858.CD002929 (http://onlinelibrary.wiley.com/doi/10.1002/14651858.CD002929/abstract). For full citation and authors’ details see reference 1.

**The full text is freely available from:** http://www.cochranejournalclub.com/masks-for-preventing-wound-infection-in-surgery/pdf/CD002929.pdf
